# Computed Tomography Scan Architectural Measurements in Adult Foot and Ankle Surgery: A Narrative Review for Orthopaedic Trainees

**DOI:** 10.7759/cureus.32039

**Published:** 2022-11-30

**Authors:** Fitzgerald Anazor, Vusumuzi Sibanda, Aisha Abubakar, Baljinder S Dhinsa

**Affiliations:** 1 Trauma and Orthopaedics, William Harvey Hospital, Ashford, GBR

**Keywords:** foot deformity, radiological findings, foot and ankle surgery, weight-bearing ct, ct scan, computed tomography scan

## Abstract

CT scan plays an important role in adult foot and ankle surgery. Plain radiographs are usually the first-line imaging modality for assessing foot and ankle bone and joint architectural abnormalities. However, despite the fact that a CT scan is more expensive and associated with higher radiation exposure, it offers better imaging quality for the assessment of bony lesions in orthopaedics and trauma. Evidence has shown that more accurate measurements can be obtained from a CT scan compared to plain radiographs. Weight-bearing multi-detection CT scanning goes the extra mile by providing a more detailed assessment, especially for intra-articular fractures, and mirrors the real-life foot and ankle dynamics compared to conventional non-weight-bearing CT scans. It also has a relatively lower radiation dose compared to conventional CT scans. CT scan is the best modality for assessing bony lesions whereas MRI is better for soft tissue pathology.

An understanding of the role of CT scan in the anatomical assessment of the foot and ankle will help improve communication between orthopaedic surgeons, radiologists, and radiographers. A thorough understanding of when to use a CT scan compared to the other imaging modalities will also lead to better surgical outcomes, reduced cost, and reduced risk from radiation exposure. This review article analyzes the role of CT in assessing relevant radiographic architectural measurements for diagnosis and surgical planning in adult foot and ankle surgery.

## Introduction and background

CT scan has a key role in adult foot and ankle surgery. Despite the fact that a CT scan is more expensive and has a higher radiation dose, it offers better imaging quality for assessing bony lesions in orthopaedics and trauma. An imaging modality that offers superior accuracy in assessing foot and ankle architecture is of utmost importance as it reduces measurement errors arising from image superimposition and distortion that occurs with plain radiographs [1]. This imaging modality has to be readily available, relatively affordable, and have low radiation doses. CT, especially with recent advances in weight-bearing cone beam technology, offers most of these advantages if well-selected. Many of the architectural measurements can initially be assessed using plain radiographs. However, a multi-detector CT scan with three-dimensional (3D) reconstruction is more accurate and more specific for selecting the treatment modality and planning for surgery [1,2].

The main role of foot and ankle CT in preoperative surgical planning involves the assessment of deformities in adults, assessment of peri-articular or intra-articular fractures, periprosthetic fractures, prosthesis loosening, and bony collapse from avascular necrosis. It is also employed in postoperative imaging to assess whether the preoperative goals were achieved. A detailed assessment of foot and ankle architectural distortions via relevant measurements leads to effective grading of disease severity in many cases, which can provide a guide for treatment modality. This also aids communication in research and helps the clinician paint a clearer picture of the problem severity when discussing with patients as part of shared decision-making.

The aim of this paper is to present the indications and relevant architectural measurements utilised for the most common foot and ankle pathologies, with a focus on orthopaedic trainees as the target audience. However, an understanding of these architectural measurements is also important for radiology trainees, trauma and orthopaedic surgeons, radiologists, and radiographers. We believe it will lead to the use of “a common language” for these measurement terms, reduce time spent on repeat imaging as there will be better communication among teams, and will also lead to more efficient multi-disciplinary team meetings, thereby promoting improved patient care. 

Imaging protocol

An ankle CT protocol is usually performed without contrast except for CT arthrography (e.g., when evaluating for suspected osteochondral lesions). The scout film is usually taken from the middle 1/3 of the leg to the heel, the patient is put in a supine position, and axial thin-cut slices for the foot or ankle protocol are usually <1.5-mm thick. The proximal extent of the scan depends on the exact pathology being evaluated. Axial images are obtained and reformatted to provide sagittal, coronal, and oblique images including a 3D reconstruction image. The CT foot protocol evaluates the forefoot and midfoot. The scout film involves the whole foot. The extent of the CT image is typically from the tip of the toes to the mid-tarsal joints but this may vary depending on the indication [3,4]. The patient is kept in a supine position, and sagittal, coronal, and axial images are obtained. Slice thickness is as for the ankle protocol. Weight-bearing foot and ankle CT scans provide a more reliable tool for radiographic measurements compared with conventional CT scans [1].

## Review

Role of CT scan in various foot and ankle orthopaedic conditions

Ankle Fractures/Dislocation Including Pilon Fractures

Preoperative CT scan is indicated in the Arbeitsgemeinschaft fur Osteosynthesefragen (AO) 44A injuries with a vertical medial malleolus fracture, AO 44B injuries with a posterior malleolus fracture, all AO 44C injuries and ankle fractures with a Tillaux-Chaput fragment [5]. Moreover, the CT scan has a significant potential to change the surgical management plan for these injuries [6]. CT scan is also important in identifying occult fractures or intra-articular osteochondral lesions in patients who have sustained high energy injuries/polytrauma, providing useful information on displacement and size. Similarly, a CT arthrogram is useful for evaluating suspected chondral and osteochondral injuries as an alternative to an MRI scan in the non-acute setting for patients with a contraindication to an MRI scan. Axial CT scan provides a more accurate assessment of the incisura and syndesmotic disruption compared to plain radiographs but is less accurate than an MRI scan [7,8]. 

**Figure 1:**
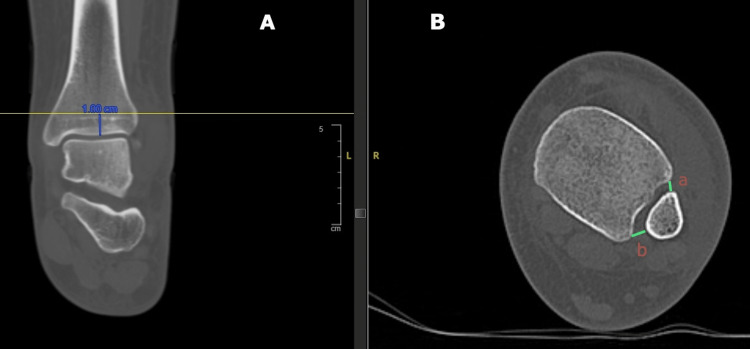
Image A is a coronal CT scan image with the corresponding axial CT scan in image B showing measurements for the distal tibiofibular syndesmosis at a level 1 cm above the tibial plafond a: anterior interval; b: posterior interval

Assessment of the syndesmosis on axial CT is performed in various ways. One method utilised by the authors is through measurements taken at a level 1 cm proximal to the level of the tibial plafond [9]. Two measurements are taken (by taking cognisance of the image magnification): the anterior interval (normal value: <4 mm) is the gap between the tip of the anterior tibial tubercle and the nearest point on the fibula and the posterior interval (normal value: <4 mm) is the gap between the lateral border of the posterior tibial tubercle and the medial border of the fibula (Figure 1) [10].

**Figure 2:**
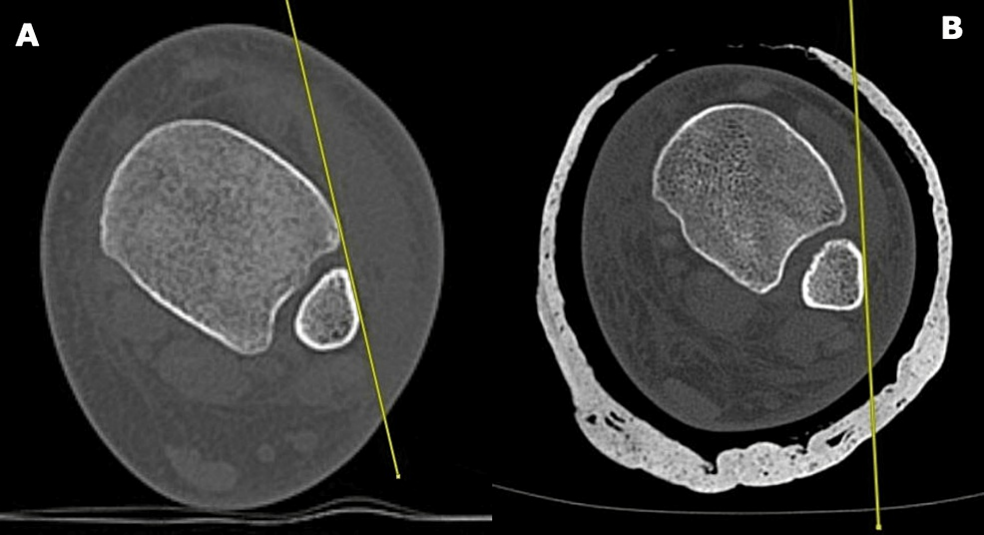
The tibiofibular line (TFL) Image A on the left shows the TFL measurement for a normal ankle while the image B on the right shows an inadequately reduced ankle syndesmosis as evidenced by the TFL

Another useful measurement parameter for assessing the adequacy of the syndesmosis reduction is the tibiofibular line (TFL). This is a tangential line traced along the anterolateral surface of the distal fibula to the distal tibia Tillaux-Chaput anterior tubercle. The distance between this line and the Tillaux-Chaput anterior tubercle should be less than 2 mm (Figure 2) [11].

CT also provides useful information during preoperative planning for posterior malleolus fractures regarding the optimal direction/trajectory for the fixation screw [12,13]. 

For distal tibia pilon fractures, a CT scan with 3D reconstruction is essential for preoperative planning following initial temporary external fixation for damage control (“span, scan, and plan”). For example, in the study by Tornetta and Gorup [14], a preoperative CT scan led to a change of the operative plan in 64% of cases. This study also showed that CT provided more information on parameters like fracture impaction, number of fragments, and severity of comminution in 82% of cases. The amount of articular step-off in intra-articular foot and ankle fractures is also best assessed on a CT scan to aid surgical decision-making. In pilon fractures, axial CT identifies the three key articular fragments during preoperative planning, viz, the medial, anterolateral, and posterolateral fragments [15]. Topliss, Jackson, and Atkins [16] identified two main family groups (“sagittal” and “coronal”) for pilon fractures on axial CT scan based on the direction of the main fracture line which runs perpendicular to the intermalleolar axis. 

Hindfoot Traumatic Injuries

**Figure 3:**
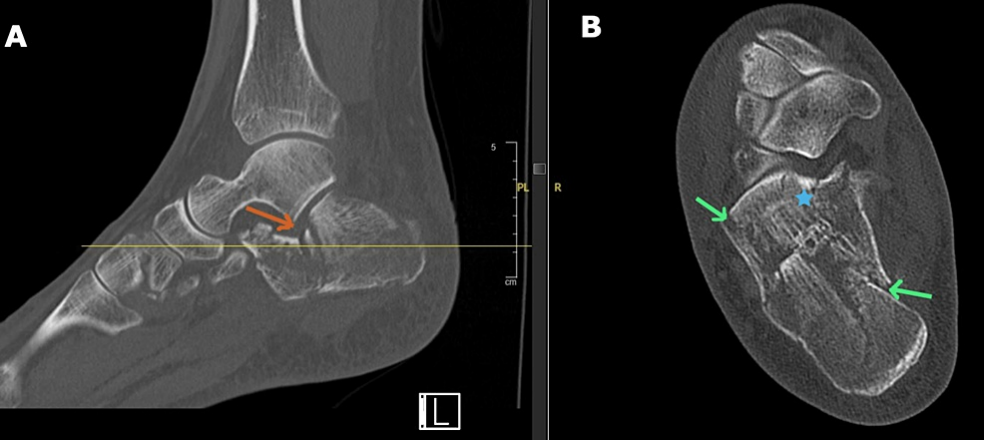
Image A is a sagittal CT image reconstruction with the orange arrow pointing to the intra-articular fracture with depression of the joint surface. The thin yellow line represents the level of the cut for the corresponding axial image on the right. Image B is an axial CT scan showing a displaced intra-articular calcaneal fracture. The green arrows point to the primary fracture line while the blue star represents the superomedial or constant fragment

A conventional CT scan is used for delineating fractures involving the calcaneum and talus. CT scan offers better visualisation of the subtalar joint compared to plain radiographs [17]. Three-dimensional CT imaging reconstruction is useful for surgical planning. Axial CT provides information on the calcaneal width and degree of heel varus. The coronal images provide information on the width and height of the calcaneus while the sagittal CT images are used for assessing the posterior facet rotational alignment and decrease in calcaneal height [18]. Bohler’s angle and the angle of Gissane can also be measured on a CT scan for assessing subtalar joint collapse in calcaneal fractures. The normal values are 20-40 degrees and 130-145 degrees respectively [19]. Axial CT scan delineating the number of articular fragments is the basis for the Sanders classification for calcaneal fractures [20]. In displaced intra-articular calcaneal fractures, the primary fracture line extends to the posterior facet and divides the articular surface into a superomedial (called the constant fragment as it is fixed firmly to the talus by the deltoid and interosseus talocalcaneal ligaments) and a superolateral fragment [21]. Secondary fracture lines exiting from the primary fracture line can also occur in more complex fractures (Figure 3).

Midfoot and Forefoot Injuries 

Multi-planar CT with 3D reconstruction is useful for detecting subtle osseous fractures including evaluation of the Lisfranc joint, especially in high-energy injuries, for the integrity of the Lisfranc ligament, amount of displacement, level of comminution, and presence of other associated forefoot and midfoot injuries. However, CT is less useful for radiological architectural assessment in low-energy foot injuries if a purely ligamentous injury rather than an osseous injury is suspected. In this scenario, an MRI scan will be more beneficial unless contraindicated [22].

Osteoarthritis and Avascular Necrosis (AVN) 

CT scan is employed as a second-line imaging modality (after plain radiographs) for assessment of the degree of deformities arising from osteoarthritis and plays a key role in surgical planning for corrective osteotomies. CT scan is also used to assess the amount of bony collapse in avascular necrosis. This includes the assessment of talar fractures for the degree/amount of collapse and fragmentation of the talar dome, which is all useful for surgical planning [23].

Deformities of the Foot 

The foot and ankle architectural distortion leading to the deformities described in this section could be idiopathic or congenital and could be secondary to sequelae of fracture mal-union, degenerative arthritis, inflammatory arthritis, Charcot arthropathy (neuropathic arthropathy), joint/bone infections, tendon deficiencies (like in posterior tibial tendon deficiency causing adult flat foot) and iatrogenic (e.g., following surgery). Apart from the assessment of intra-articular fractures for trauma, deformity assessment is one area where a CT scan plays a very important role. Just like plain radiographs, conventional CT scans can be used to assess the severity of deformities through measurement of the relevant angles (e.g., in hallux valgus and pes planus). CT, however, offers a better assessment of the degree of articular surface coverage (e.g., talonavicular coverage in pes planus), provides better detail about associated occult stress fractures, and the degree of osteoarthritis, which can alter the surgical treatment plan.

Despite some of these advantages, conventional CT has the same problem as plain radiographs in relation to the absence of weight-bearing. This problem can be eliminated via the use of weight-bearing cone beam CT technology, which provides a true 3D image rendering of the foot in weight-bearing and provides lower radiation doses. For example, in relatively common foot deformities like hallux valgus and pes planus, weight-bearing CT is used to measure various angles. These radiographic angles, including their normal values and clinical applications, are summarised in Table 1 [24-30].

**Table 1:** Summary of some of the key measurements in adult foot and ankle deformity assessment* *[24-30]

CT image reconstruction	Measurement/angle	Description	Normal value	Clinical relevance
Axial CT hindfoot	Talus-1st metatarsal angle (Simmons angle)	The angle between the axis of 1st metatarsal and the axis of the talus	~7 degrees	Usually, >16 degrees in adult-acquired flat foot/pes planus
Axial CT hindfoot	Talo-calcaneal angle (Kite's angle)	The angle formed between a line bisecting the head/neck of the talus and another line tangential to the lateral border of the calcaneus	25-40 degrees	>40 degrees in hindfoot valgus (e.g., in pes planus), <25 degrees in hindfoot varus (e.g., in club foot)
Axial CT hindfoot	Calcaneocuboid angle	The angle between tangential lines drawn along the lateral border of the cuboid and the lateral border of the calcaneus	0-5 degrees	Increased in flat-foot deformity (due to forefoot abduction). Decreased in clubfoot
Axial CT midfoot	Talonavicular uncoverage angle	The angle between lines parallel to the articular surfaces of the navicular and that of the talar head	15-20 degrees	Adult-acquired flat foot, >20 degrees. Assesses the degree of talonavicular subluxation
Axial CT midfoot	Talonavicular uncoverage	Percentage of the talar head articular surface not covered by the navicular in relation to the total extent of the talar head articular surface	Normal talonavicular coverage is 75-100%	>40% uncoverage in pes planus
Axial CT forefoot	Hallux valgus angle (HVA)	The angle between the mid-axial line of the 1st metatarsal shaft and the mid-axial line of the big toe proximal phalanx	<15 degrees	>15 degrees in hallux valgus deformity
Axial CT forefoot	Hallux valgus intermetatarsal angle (IMA)	The angle between the mid-axes of the 1st and 2nd metatarsals	<9 degrees	IM >10 degrees in hallux valgus deformity
Axial CT forefoot	Hallux valgus interphalangeal angle	The angle between the diaphyseal axial and metaphyseal axial lines of the proximal phalanx of the big toe	<15 degrees	>15 degrees in hallux valgus
Axial CT forefoot	Distal metatarsal articular angle (DMAA)	The angle between the articular surface of the head and the shaft of the 1st metatarsal	<10 degrees	DMAA >10 degrees in hallux valgus deformity
Axial CT forefoot	Metatarsus adductus angle (MAA)	The angle between a line in the axis of the 2nd metatarsal and a perpendicular line to the axis of the 4th or 5th metatarsocuboid joint	<15-20 degrees	>25 degrees in metatarsus adductus deformity
Sagittal CT	Calcaneal pitch	The angle between the line parallel to the inferior calcaneal surface and the horizontal plane	20-30 degrees	Usually, <18 degrees in pes planus
Sagittal CT	Talus-1st metatarsal angle (Meary's angle)	The angle formed between the mid-talus axial line and the mid 1st metatarsal axial line	0 degrees	>4 degrees with the angle apex pointing superiorly in pes planus; >4 degrees with the angle apex pointing inferiorly in pes cavus
Sagittal CT	Effective 1st metatarsal length	The distance between 2 lines projected down from each end of the 1st metatarsal		Excessive 1st metatarsal length in comparison to that of the 2nd metatarsal seen in hallux valgus

**Figure 4:**
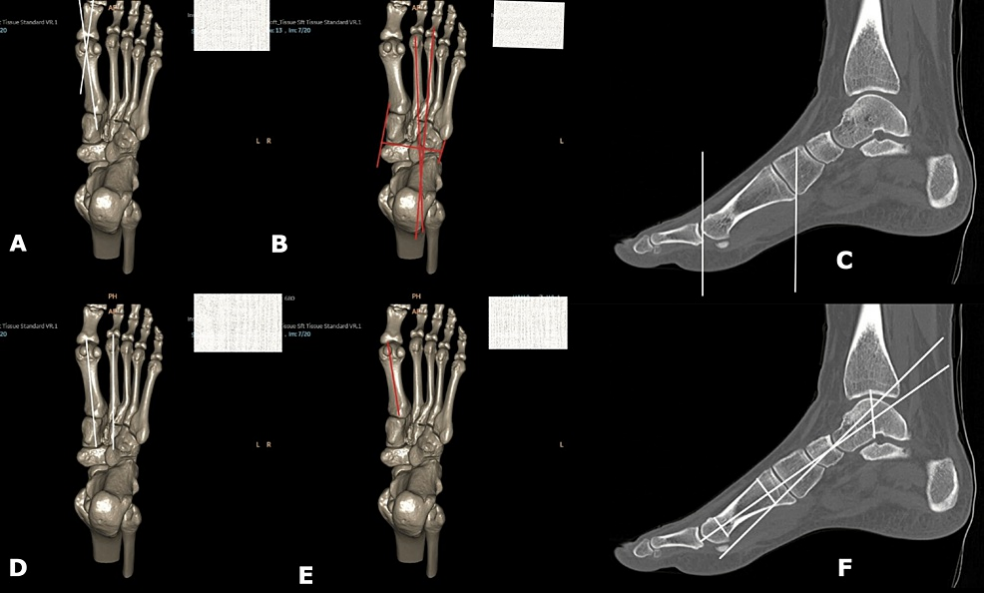
Three-dimensional reconstructed CT images (panes A, B, D, and E) and sagittal reformatted CT images (panes C and F) showing measurements for (a) hallux-valgus angle, (b) metatarsus adductus angle, (c) effective 1st metatarsal length, (d) intermetatarsal angle for hallux valgus, (e) absolute 1st metatarsal angle, and (f) Meary's angle

CT scan images showing some of the relevant measurements for the assessment of foot deformities are depicted in Figure 4.

Neuropathic Arthropathy 

Neuropathic arthropathy (Charcot arthropathy) deserves special consideration due to the multiple complex foot and ankle deformities that can arise from the condition. It has varying etiologies and is a major cause of foot and ankle architectural distortions leading to various deformities. Plain radiographs remain the initial imaging of choice in suspected Charcot neuroarthropathy but the findings compared to CT scans vary depending on the stage of the disease.

In Charcot arthropathy, the modified Eichenholtz classification divides the disease into stage 0 (no change on plain radiographs), stage 1 (development stage characterised by fragmentation, joint subluxation, and dislocation), stage 2 (coalescence stage with debris resorption and bone fragment fusion), and stage 3 (remodeling characterised by deformity consolidation and joint arthrosis) [31]. Plain radiographs are usually normal in “stage 0”. Conventional CT has a limited role in architectural assessment in stage 0 and MRI and/or bone scan is more useful to distinguish the typical features from edematous changes due to microfractures or infection [32]. CT, just like plain radiographs, will show features of bone dissolution, fragmentation, and joint subluxation/dislocation in stage 1 Charcot arthropathy. Another radiographic feature of this stage is the flattening of the metatarsal heads. By stage 3 disease, there may be midfoot collapse resulting in rocker bottom foot and forefoot abduction deformities. These Charcot arthropathy-induced foot/ankle deformities can be assessed by measuring the calcaneal pitch, Meary's angle, axial talocalcaneal angle, and other deformity assessment parameters as described previously in Table 1. CT scan offers better images for assessing these deformities, especially for reconstructive surgical planning.

Postoperative Foot and Ankle Imaging 

CT is used for evaluating postoperative ankle pain following open reduction and internal fixation (ORIF) for ankle fractures if there is suspected mal-reduction of the distal tibiofibular syndesmosis as described previously. This mal-reduction, as seen on postoperative ankle CT, is seen in approximately 52% of cases [33]. To reduce the incidence of this problem, appropriate preoperative planning and/or intraoperative CT may be employed. CT will also reveal subchondral intra-articular screw penetration, which can be an etiology for postoperative pain and may not be readily visible on plain radiographs. In addition, CT is useful for evaluating secondary deformities arising from mal-union if plain radiographs do not yield all the information required. CT is occasionally used postoperatively for evaluating the adequacy of corrective osteotomies, and the assessment of the degree of postoperative traumatic osteoarthritis and chronic neuroarthropathy.

In total ankle arthroplasty, CT offers earlier detection and better assessment of the degree of implant loosening, implant displacement, periprosthetic fracture displacement, and quantification of the degree of early osteolysis [34]. Attenuation protocols may be necessary to reduce metal-induced artifacts for postoperative CT imaging when an implant or prosthesis is present. In addition, weight-bearing CT scan post-ankle arthroplasty can be used to assess for proper prosthesis alignment. 

## Conclusions

This article demonstrates that most of the foot and ankle architectural measurements traditionally assessed on plain radiographs can be assessed on CT scans as well. It is important to educate the new generation of orthopaedic doctors about the role of appropriate imaging and moving on from the older ideas of “CT is just going to waste time” and “we can see everything on plain radiographs”. Although CT is more expensive and has relatively higher radiation doses, it plays a major role in accurate deformity assessment and intra-articular fracture delineation in adults when compared to plain radiographs. Weight-bearing cone beam CT technology with lower radiation doses reduces the radiation risks associated with conventional CT scans. Weight-bearing cone beam CT is available in our hospital and its availability in the United Kingdom is improving. Nevertheless, weight-bearing plain radiographs are still useful in certain scenarios, e.g., when assessing ankle fractures for talar shift in isolated lateral malleolar Weber B fractures.

An understanding of the role of CT scan in anatomical assessment of the foot and ankle either preoperatively, intraoperatively, or postoperatively will help improve communication between orthopaedic surgeons, radiologists, and radiographers. Selection of the appropriate imaging modality also serves to reduce cost, unnecessary radiation exposure, and errors in surgical operations. Sound knowledge of the indications for foot and ankle CT scans, their pros and cons, and the architectural measurements that can be obtained will lead to more effective use of this imaging modality.
